# Efficient Allocation for Downlink Multi-Channel NOMA Systems Considering Complex Constraints

**DOI:** 10.3390/s21051833

**Published:** 2021-03-06

**Authors:** Zhengjia Xu, Ivan Petrunin, Teng Li, Antonios Tsourdos

**Affiliations:** School of Aerospace, Transport and Manufacturing, Cranfield University, Bedford MK43 0AL, UK; i.petrunin@cranfield.ac.uk (I.P.); tengli@cranfield.ac.uk (T.L.); a.tsourdos@cranfield.ac.uk (A.T.)

**Keywords:** resource allocation, heuristic solution, NOMA, greedy policy

## Abstract

To enable an efficient dynamic power and channel allocation (DPCA) for users in the downlink multi-channel non-orthogonal multiple access (MC-NOMA) systems, this paper regards the optimization as the combinatorial problem, and proposes three heuristic solutions, i.e., stochastic algorithm, two-stage greedy randomized adaptive search (GRASP), and two-stage stochastic sample greedy (SSD). Additionally, multiple complicated constraints are taken into consideration according to practical scenarios, for instance, the capacity for per sub-channel, power budget for per sub-channel, power budget for users, minimum data rate, and the priority control during the allocation. The effectiveness of the algorithms is compared by demonstration, and the algorithm performance is compared by simulations. Stochastic solution is useful for the overwhelmed sub-channel resources, i.e., spectrum dense environment with less data rate requirement. With small sub-channel number, i.e., spectrum scarce environment, both GRASP and SSD outperform the stochastic algorithm in terms of bigger data rate (achieve more than six times higher data rate) while having a shorter running time. SSD shows benefits with more channels compared with GRASP due to the low computational complexity (saves 66% running time compared with GRASP while maintaining similar data rate outcomes). With a small sub-channel number, GRASP shows a better performance in terms of the average data rate, variance, and time consumption than SSG.

## 1. Introduction

**Figure 1:**
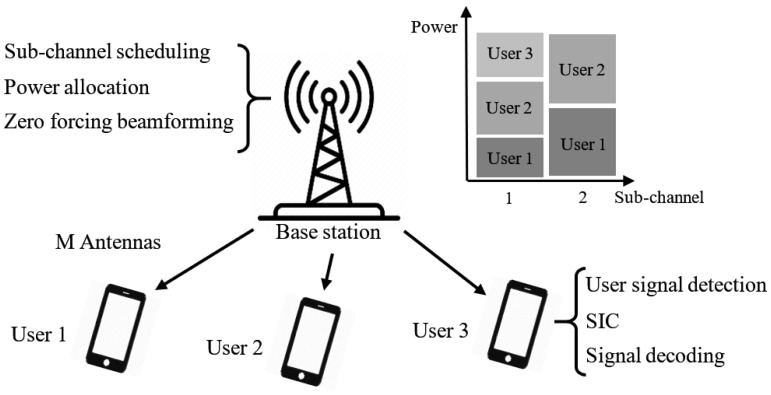
Illustration of downlink MC-NOMA system.

To fully exploit spectrum resources and meet the explosive traffic growth, the non-orthogonal multiple access (NOMA) scheme has attracted sufficient attention in recent years for the next-generation cellular systems, and envisions to offer significant improvement for the current communication structures [1]. Distinct from orthogonal multiple access (OMA) schemes (e.g., orthogonal frequency division multiple access (OFDMA) and single-carrier FDMA (SC-FDMA)) based communication system (e.g., long-term evolution (LTE)), NOMA allows the sharing of spectrum in the time and frequency domain among multiple users simultaneously at the cost of increasing computational complexity in detections at the receiver side. The NOMA scheme provides the possibility of outperforming OMA in dense networks with large numbers of users [2]. Some NOMA relevant applications are investigated, e.g., ensuring the multiple power beacons (PBs) based NOMA-mobile edge computing (MEC) to transmit in a reliable and energy-efficient way [3] and applying NOMA and energy harvesting to support device-to-device (D2D) transmission [4]. The NOMA construction is enabled with the successive interference cancellation (SIC) which has a pre-known knowledge-base of users before allocation [2]. An illustrative diagram of NOMA communication is presented in Figure 1. Therefore, an efficient joint resource allocation of spectrum frequency and transmit power, i.e., joint dynamic power and channel allocation (DPCA) [5], is dominant to form the NOMA concept so as to improve the efficiency in wireless communication.

The DPCA problem is typically regarded as a function optimization problem (FOP) and solved with the joint optimization of scheduling and power control [6]. Cui et al. [7] utilized the matching theory and successive convex approximation techniques for tackling the user scheduling and power allocation problems. Wei et al. [8] proposed a reliable allocation algorithm for multi-channel NOMA (MC-NOMA) considering the uncertainty and imperfect of channel state information (CSI). Fang et al. [9] exploited the user scheduling and power allocation with imperfect CSI by propositions of iterative algorithms, where the outage probability requirement, minimum user data rate, and user capacity per sub-channel are specified in modeling the allocation. The classical mixed integer formulations solved by searching, matching, and iterative algorithms for addressing DPCA are commonly believed to have a large number of iterative steps when obtaining the convergence results [5] due to the continuous variable of power budget, which drives demands of exploring solutions with the low running cost.

Dai et al. [2] compared Welch-bound equality spread multiple access (WSMA) based NOMA communication with OMA scheme leading to the conclusion of reducing the implementation complexity. For releasing the computations in the allocation, Liu and Petrova [5] proposed a water-filling based algorithm for allocating the power among inter-channels. Nain et al. [10] proposed a method that removes users who do not satisfy the derived condition before the allocation using the paring and Lagrangian approach. Fang et al. [11] applied convex relaxation and dual-decomposition techniques to release the computation loads in the optimization, where the optimal closed-form power allocation expressions were derived with the Lagrangian approach.

The other idea of relaxing the computation when the power variable involved is implemented by splitting the continuous power into a finite set of power streams during processing. Sokun et al. [12] considered the discretized the power and resource block allocations, and proposed an iterative sub-optimal heuristic for constructing the energy-efficient orthogonal frequency division multiple access (OFDMA)-based wireless communication system. D’Andreagiovanni et al. [13] sampled the power resources and construct a single knapsack polytope with two GUB inequalities for designing the wireless network, where the user transmitters are assumed to operate in the same frequency.

Differently from the exact algorithms, the heuristic solution presents more advantages in the large networks due to its efficiency, especially integrated with the approximation strategy for relaxing the constraints. However, the heuristic solution performance is typically not provable and unable to provide certain quality. D’andreagiovanni et al. [14] integrated the variable fixing with mixed integer programming (MIP) heuristic algorithm guided by linear programming (LP) relaxations in the constraints to address a robust optimization model. Capone et al. [15] relaxed the minimum signal-to-noise-and interference ratio (SINR) constraint to a knapsack constraint aiming at approaching the global optimality.

Several fundamental works have been done on modeling diverse DPCA problems and investigating NOMA performance. The user fairness is modeled in [16] to balance the user performance during allocations through adding the user sum weights in the sum rate function. Lei et al. [17] modeled the maximum user number per sub-channel as a constraint in DPCA for practical consideration, as well as considering the processing delay in the SIC. The diverse transmission power budget per individual sub-channel is modeled as a constraint in [5]. To guarantee the weak users’ QoS, a W-QoS-based NOMA algorithm is proposed in [18] which envisions to be merged with the established selective and clustering methods, and guarantees the NOMA rate higher than the TDMA rate.

Submodularity is a useful tool for modeling the objective functions for the resource allocation problems. It simply requires that the marginal gain value obtained by allocating a resource diminishes as the number of allocated sources increases. Finding the optimal solution of submodular maximization is proven to be NP-hard [19], which implies that the complexity of the problem explodes as the problem size increases. Greedy-based algorithms have been widely applied in submodular maximization problems due to the efficiency and capability to provide a constant factor of approximation ratio. Typical applications include Max-Cut [20], facility location selection [21,22], sensor placement [23,24,25], and machine learning [26,27,28]. Recently, greedy-based algorithms have shown great potentials in reducing computational complexity when handling resource allocation problems [29,30].

This paper considers the allocation problem as the combinatorial problem by way of discretizing continuous power resources into finite sets based on the sampling concept. Three typical heuristic solutions are proposed owing to efficiency and ease of difficulty in modeling, i.e., stochastic strategy, greedy randomized adaptive search (GRASP), and stochastic sample greedy (SSG) strategy. Distinct from the non-convex problem based solvers, the applied combinatorial solver does not require searching of optimal results which accelerates the computation. Distinct from other greedy based solvers [31,32], the randomization of the sub-channel allocation and the resource assignment are integrated, as well as the consideration of multiple practical constraints.

The contributions of this paper are: (1) We propose greedy enabled low-complexity algorithms useful for the general environments (extreme spectrum dense and scarce environments are included), where DPCA is regarded as the combinatorial problem. (2) To reflect the practical scenario, we introduce some feasibility constraints (e.g., user number limit per each sub-channel, maximum power restrictions among users and BSs, and the minimum data rate per user) as the hard constraints which must be ensured during allocations. The priority control management is represented as a soft constraint modeling that the function is taken into account while not needing to be guaranteed for each iterative. (3) Some strategies are presented for the exclusive addressing of DPCA. For instance, a two-phase allocation structure is applied to maintain the hard constraint of the minimum data rate requirement, the user capacity is achieved through the construction of the objective function, and the sampling of power values is applied for reducing the computational complexity in the combinatorial problem.

This paper organizes as follows. Section 2 discusses typical greedy algorithm along with its worst performance. Section 3 presents the formulation of the DPCA problem that is considered in this paper. Section 4 proposes heuristic solution algorithms for solving the DPCA problem. The performance of the proposed algorithms is testified in Section 5 through simulations. Section 6 offers the conclusion of the contributions in this paper.

## 2. Preliminary Background

This section discusses characteristics in the unified greedy algorithms (see Algorithm 1) for addressing the allocation problems with single objective function.
**Algorithm 1** Greedy algorithm subject to matroid constraints**Input:**$f:2^{N}\to R_{\geq 0},N,I$ **Output:** A set S⊆I
1:S←∅2:**while**∃u∈N such that S∪{u}∈I **do**3:    $u^{*}\leftarrow \underset{u\in N\backslash S}{\arg\;\max}f\left(u|S\right)$4:    $S\leftarrow S\cup \{u^{*}\}$5:**end while**6:**return***S*


Since the greedy policy essentially adopts iterative allocation, we denote *u* as the current resource of interest and *Z* as the allocated set. The objective function with the *u* resource denotes f(u|Z) and aims to be maximized. Consequently, the differential objective function Δf(u|Z) can be calculated by using the marginal gain value (*mgv*) [33]:

**Definition** **1** (Marginal gain value (*mgv*))**.** *For a set function $f:2^{N}\to R$, a set Z⊂N, and an element u∈N, define the mgv of u given Z as*

Δf(u|Z)≐f(Z∪{u})−f(Z),

*where N is named as the ground set which is a finite set. The notation “≐” means equal by definition.*

The objective function considered in this work is submodular and monotone. The formal definitions of submodularity and monotonicity are provided in the following.

**Definition** **2** (Submodularity [34])**.** *A set function $f:2^{N}\to R$ is submodular, if ∀X,Y⊆N,*

f(X)+f(Y)≥f(X∩Y)+f(X∪Y),

*where N is the ground set. N, X, and Y are finite sets.*

Equivalently, the submodularity can also be derived by combining Definitions 1 and 2 as:

(1) Δf(u|X)≥Δf(u|Y).∀X⊆Y⊆N,u∈N\Y.

Equation (1) is also named as the diminishing return [34], which is an important property for submodular functions. Intuitively, the marginal gain value when adding a new element *u* to a determined set increases with a smaller set size.

**Definition** **3** (Monotonicity [33])**.** *A set function $f:2^{N}\to R$ is monotone, if ∀X⊆Y⊆N, then f(X)≤f(Y). f is non-monotone if it is not monotone.*

The constraint that one resource can be allocated to at most one user is represented by the partition matroid constraint. The definition of a matroid is described as:

**Definition** **4** (Matroid [35])**.** *A matroid is a pair M=(N,I) where N is the ground set and $I\subseteq 2^{N}$ is a collection of independent sets, satisfying:*

- ∅∈I
- if X⊆Y,Y∈I, then X∈I
- if X,Y∈I,|X|<|Y|, then ∃u∈Y\X such that X∪{u}∈I.

*|·| denotes the cardinality operation.*

Given the above definitions of *mgv*, submodularity, monotonicity, and matroid, a partition matroid constraint can be used for formulating the maximization function, where the partition matroid denotes that an independent subset *S* contain at most a certain number of elements from each of the disjoint partitions of N. Consequently, the worst performance of the typical greedy algorithm performance is denoted:

**Theorem** **1.** *The Greedy algorithm achieves an approximation ratio of at least 50% of optimality for maximizing monotone submodular functions subject to matroid constraints.*

**Proof.**  Let $OPT\backslash S=\{a_{1},\cdots ,a_{l}\}$ where OPT is the optimal solution. Let $a^{*}$ denote the element selected by the greedy algorithm at step *j* given $S_{j-1}$. Since $a^{*}$ is optimal with the greedy concept, we then have

$$f\left(S_{j-1}\cup \left\{a_{j}\right\}\right)-f\left(S_{j-1}\right)\leq f\left(S_{j-1}\cup \left\{a^{*}\right\}\right)-f\left(S_{j-1}\right)$$

When the objective function is monotone, the optimality is obtained:

$$\begin{array}{ccc} f(OPT) & \leq f(S\cup OPT) & \left(\mathrm{monotonicity}\right)\\ \; & =f\left(S\right)+\sum_{j=1}^{l}\left(f\left(S\cup \left\{a_{1},\cdots ,a_{j}\right\}\right)-f\left(S\cup \left\{a_{1},\cdots ,a_{j-1}\right\}\right)\right) & \\ \; & =f\left(S\right)+\sum_{j=1}^{l}\left(\Delta a_{j}|f\left(S\cup \left\{a_{1},\cdots ,a_{j-1}\right\}\right)\right) & \end{array}$$

Then, by submodularity, we get

$$\begin{array}{cc} f(OPT) & \leq f\left(S\right)+\sum_{j=1}^{l}\Delta f\left(a_{j}|S\right)\\ \; & \leq f\left(S\right)+\sum_{j=1}^{l}\Delta f\left(a_{j}|S_{j-1}\right)\\ \; & \leq f\left(S\right)+\sum_{j=1}^{l}\Delta f\left(a^{*}|S_{j-1}\right)\\ \; & \leq f\left(S\right)+\sum_{j=1}^{r}\Delta f\left(a^{*}|S_{j-1}\right)\\ \; & =2f(S) \end{array}$$

where r=|OPT|.    □

It is worth noting that, when the hard constraints are mitigated (such as in the spectrum dense environment), the greedy algorithm used in this paper can be regarded as a simplified version with the typical greedy algorithm format, and hence the 50% performance degradation applies.

## 3. Problem Formulation

To address the DPCA problem, the user sequence and power sequence are the outcomes to obtain the solution that users and their transmission power are allocated to the individual sub-channel.

For the downlink MC-NOMA system [5], we suppose that m∈M(1,2,3,⋯,M) users are allocated with n∈N(1,2,3,⋯,N) sub-channels in the base station (BS). The allocated sub-channels form a sub-channel set l∈L(1,2,3,⋯,L), where L⊂N. We define U as the allocated user sequence set, where the element in U is U∈U and U⊂M. The power budget for the *m*th user is defined as $w_{m}\in W$ where W is the power budget set for users representing the maximum power output for each transmission. The power budget for the *n*th sub-channel is defined as $k_{n}\in K$ with a determined power budget set K. The overall power budget for all the *N* sub-channels thus denotes $K_{x}=\sum_{i=1}^{N}k_{i}$. We define the power sequence set P which is treated as the discrete variable through the sampling strategy to construct the combinatorial optimization problem. For the user sequence U, the allocated cumulative power *p* over *n*th sub-channel is defined as $p_{U(n)}$ and can be obtained by $p_{U(n)}=\sum_{i=1}^{r_{n}}p_{U_{i}\left(n\right)}$. Similarly, $p_{U(m)}$ denotes the allocated cumulative power for the *m*th user and $p_{U(m)}=\sum_{i=1}^{L}p_{U_{i}\left(m\right)}$.

The instantaneous channel quality indicator (CQI) [5] defined as ϕ∈Φ is used as the SINR for representing the effects from the channel environment. $\phi_{U(n)}$ represents the CQI for the allocated user *U* over the *n*th sub-channel. The channel propagation effects, such as small-scale fading, large-scale fading, and Doppler effect, are reflected in CQI, while they are not modeled in this paper.

We assume that the inter-cell interference [36] induced by multiplexing of multiple users over the same spectrum is resolved by SIC. The instantaneous data rate *D* for the allocated user *U* over *n* sub-channel is estimated by Shannon’s theorem:

(2) $$D_{U(n)}=f\left(P\right)=Blb\left(1+p_{U(n)}\phi_{U(n)}\right)$$

where *B* denotes the bandwidth of the sub-channel and assumes to be equivalent among all the sub-channels. The unit of the data rate is nat/s.

The cumulative data rate $\hat{D}$ is obtained by summarizing the data rate *D* of individual user *m* over *N* sub-channels, and it is denoted:

(3) $${\hat{D}}_{m}=\sum_{i=1}^{L}D_{U_{i}\left(m\right),i},i\in L$$

Consequently, the general DPCA problem can be solved by optimizing the maximum data rate for the allocated user set U:

(4) $$\begin{array}{c} \max F=\sum_{m\in M}{\hat{D}}_{m} \end{array}$$

This paper considers several feasibility constraints and useful functions in practice, i.e., user number limit per each sub-channel, maximum power restrictions among users and BSs, the minimum data rate per user, and the user priority control during allocation. Among them, the former three feasibility constraints are treated as the hard constraint, whereas the priority control function is the soft constraint to relax the computation as it is not necessarily imposed for each allocation. This paper aims at maximizing the cumulative data rate with the discrete unit power cost sampled in advance. The overall DPCA problem is exploited by solving the combinatorial optimization problem extended from the (4) format, and is reformulated as below:

(5) $$\begin{array}{cc} \; & \max F=\sum_{m\in M,n\in N,p\in P}{\bar{D}}_{m}\left(m,n,p\right)\\ & {\bar{D}}_{m}\left(m,n,p\right)=\beta_{m}\sum_{i=1}^{N}D_{U_{i}\left(m\right),i}\left(p\right),\\ & s.t.\;C1:r_{n}\leq s_{n},s_{n}\in S,\forall n\in N\\ & \;\;\;C2:0\leq p_{U(n)}\leq k_{n},k_{n}\in K,\forall n\in N,\\ & \;\;\;C3:0\leq p_{U(m)}\leq w_{m},w_{m}\in W,\forall m\in M,\\ & \;\;\;C4:{\hat{D}}_{m}\geq q_{m},q_{m}\in Q,\forall m\in M. \end{array}$$

where the P are regarded as the discrete variable in the proposed solutions.

As depicted in (5), we develop the following constraints when constructing the objective function: (1) The maximum user number $r_{n}=len\left(U_{n}\right)$ multiplexed per individual sub-channel is limited to a certain number $s_{n}\in S$ [37] (referring to the first inequality Constraint C1), where the len function calculates the length of the sequence. (2) The power budget among sub-channels $p_{U(n)}$ is limited to a maximum power budget $k_{n}\in K$ for ∀n∈N to prevent the over-usage of single sub-channel (referring to the second inequality Constraint C2), where $p_{U(n)}=\sum_{i=1}^{r_{n}}p_{U_{i}\left(n\right)}$. (3) Given the maximum power budget among users, the allocated cumulative power $p_{U(m)}$ for the *m*th user is limited to a diverse power budget per each user $w_{m}\in W$ over ∀m∈M (referring to the third inequality Constraint C3), where $p_{U(m)}=\sum_{i=1}^{L}p_{U_{i}\left(m\right)}$. (4) For ensuring the minimum QoS requirement, we apply the fourth inequality constraint, i.e., Constraint C4 to guarantee the minimum data rate among users. (5) The priority control during allocation matters due to the multi-level feature of user qualities in service. This function is developed by adding weights β in the cumulative data rate $\hat{D}$ function (3), i.e., $\beta_{m}{\hat{D}}_{m}$ in the second equation.

The defined objective function also reveals the submodularity and monotonicity according to discussions in Section 2. It is worth noting that the presented DPCA is difficult to solve with the off-the-shelf algorithms. This paper presents heuristic solutions to address this problem, and the solvers are discussed in the following section.

## 4. Heuristic Solutions

In this paper, we consider two cases: (1) only the minimum resources exist in BS for maintaining the least data rate among users (see the Theorem 2), which means that users may not be allocated with sufficient power and sub-channels to maximize the data rate; and (2) the BS resources are sufficient to achieve the maximum data rate for all the users (see the Theorem 3).

**Theorem** **2.** *To successfully enable the DPCA with the minimum constraints of data rate, the overall power budget $K_{x}$ needs to satisfy the condition:*

(6) $$\begin{array}{c} K_{x}\geq K_{max}=M\frac{e^{\frac{Q_{max}}{B}}-1}{\Phi_{min}} \end{array}$$

**Proof.**  The transmission power for the single user can be estimated from the Shannon’s theorem, i.e., $p=\frac{e^{\frac{D}{B}}-1}{\phi }$. Given the diversity in D∈Q and ϕ∈Φ, the optimal transmission power for the single user is $p_{max}=\frac{e^{\frac{Q_{max}}{B}}-1}{\Phi_{min}}$. For ease of expression, the power budget can be configured to be $M\times p_{max}$ for mitigating the computation loads, hence the maximum *K* value is obtained.    □

**Theorem** **3.** *The overall power budget $K_{x}$ needs to satisfy inequality $K_{x}\geq \sum \left(W\right)$ to enable the maximum data rate constrained to the power limit $p_{U(m)}\leq w_{m}$:*

Given the fact that the $K_{x}$ value in Theorem 3 outperforms the $K_{x}$ value in Theorem 2, the following relationship between Q, Φ, and W is obtained:

(7) $$\begin{array}{cc} \; & E\left[p_{U}\right]\leq E\left[w\right]\\ & \frac{e^{\frac{mean(Q)}{B}}-1}{mean(\Phi )}\leq mean\left(W\right)\\ & e^{\frac{mean(Q)}{B}}\leq mean\left(\Phi \right)mean\left(W\right)+1 \end{array}$$

where the mean function denotes the average values for the sets.

It is worth noting that the constraints of the maximum power budget in sub-channels K and the maximum user number S affect the allocated sub-channel sequence length *L*. Therefore, the sub-channel number is assumed to guarantee the minimum sub-channel number, i.e., the division of the maximal power budget by the minimal power budget among sub-channels.

**Remark** **1.** *For maintaining the complex constraints, the sub-channel number N is assumed to satisfy the condition:*

(8) $$\begin{array}{cc} N>L_{max} & =\frac{K_{max}}{\min(K_{min},K_{min}S_{min})}\\ & =M\frac{e^{\frac{Q_{max}}{B}}-1}{\Phi_{min}\min\left(K_{min},W_{min}S_{min}\right)} \end{array}$$

*where the min(x,y) function returns the minimum value of x and y inputs.*

Given the difficulty in addressing Cases (1) and (2) under the complex constraints with current solutions, the heuristic solutions are presented to accelerate the computation for DPCA in the following section, i.e., stochastic strategy, GRASP strategy, and SSG strategy.

### 4.1. Stochastic Strategy

The stochastic strategy based solver addresses DPCA by generating random values or sequences, i.e., the allocated sub-channel set L, the user sequence set U, and the corresponding user power set P. It is worth noting that the consideration of fairness fitting can be negligible by using the stochastic strategy, since the allocations among users are treated in equal. The optimization function (4) holds. If the β sequence follows a decreasing order, the priority control function for *M* users is reflected as a condition statement $sort\left(\hat{D}\right)==\hat{D}$ where sort is the re-ordering function. We assume $s_{n}\leq k_{n},\forall n\in N$ to reflect user number limit among sub-channels. Therefore, the stochastic strategy based DPCA solver is presented in Algorithm 2.
**Algorithm 2** Stochastic algorithm for DPCA  1:Initialization of M, N, K, Φ, S, Q, and β. $Kr_{N}=K$. $Wr_{M}=W$.  2:**while**flag==0**do**  3:    Generate random L ranging in [1,N] with the sub-channel number ranging in $\left[L_{max}+1,N\right]$.  4:    For the L set, generate numerical random set U with values ranging in [1,M] and number ranging in [1,min(S,M)].  5:    **while** i∈[1,r(n)],∀n∈L **do**  6:        Generate random power value for $P_{n,i}$ with values ranging in $\left[0,\min\left(Kr_{n},Wr_{m}\right)\right]$.  7:        $Kr_{n}=Kr_{n}-P_{n,i}$, $Wr_{m}=Wr_{m}-P_{n,i}$.  8:    **end while**  9:    **if** $sort\left(\hat{D}\right)==\hat{D}$ **then** // sort $\hat{D}$ in the ascending order when $\beta_{i+1}>\beta_{i}$.10:        ${\mathrm{flag}}_{\beta }=1$.11:    **end if**12:    **if** ${\hat{D}}_{m}\geq q_{m},\forall q\in Q$ **then**13:        ${\mathrm{flag}}_{q}=1$.14:    **end if**15:    $flag={\mathrm{flag}}_{\beta }\times {\mathrm{flag}}_{q}$.16:**end while**17:**return**L, U and P

The complicated constraints are taken into consideration. For instance, the maximum user number is reflected in Step (6); the sub-channel power budget and cumulative power budget are implemented in Step (8) by updating the remaining power budgets Kr and Wr for *K* and *W*, respectively; and the constraints of the minimum data rate and priority control are achieved by using ${\mathrm{flag}}_{q}$ (Step (14)) and ${\mathrm{flag}}_{\beta }$ (Step (12)), separately.

The expectation for $\hat{D}$ can be estimated as following:

(9) $$\begin{array}{cc} E[\hat{D}] & =\frac{f^{-1}\left(E\left[L\right]\cdot E\left[P\right]\right)}{M} \end{array}$$

where the average sub-channel number $E\left[L\right]=\frac{L_{max}+N+1}{2}$, and the expectation of data rate per sub-channel denotes:

(10) $$E\left[P\right]\approx \min(mean\left(K\right),\frac{mean(W)(1+mean(S))}{2})$$

### 4.2. Greedy Randomized Adaptive Search

Given the fact that typical greedy algorithm is only applicable for solving the single objective optimization (DPCA in this paper is a joint optimization problem), greedy randomized adaptive search (GRASP) is used and modified owing to its efficiency for combinatorial optimization problems. Providing the continuity in power candidates among sub-channels, the discretization of power value is prominent to mitigate the computational complexity as well as simplifying the local-search procedure in obtaining the local optimal outcomes.

The typical GRASP consists of two phases, i.e., a construction phase and a local search phase. The construction phase aims at selecting a preferable outcome using the randomized adaptive greedy strategy. Based on the selected outcome, the local search phase is for improving the performance at a cost of computational complexity. It is worth noting that the local search can be both added to the GRASP and SSG, thus it is not presented for the fairness comparison purpose.

Due to the low-bound constraint, i.e., the minimum data rate limit for users, a two-stage GRASP (construction phase combined) is presented. For the first stage, the minimum data rate is sufficiently allocated to users. Afterwards, the remaining power and sub-channel resources are assigned with the best performance purpose in the second stage.

Given the optimal selection for each iterative, we reformulate the objective function from Equation (5) as:

(11) $$\begin{array}{cc} F_{m,n} & \left(p,s\right)=\left\{\begin{array}{cc} \beta_{m}D_{m,n}\left(p\right){\hat{p}}_{m,n}\lambda^{{\hat{p}}_{m,n}}\; & \mathrm{if}\;0\leq r_{n}\leq s_{n}\\ 0\; & \mathrm{if}\;r_{n}>s_{n} \end{array}\right.\\ & {\hat{p}}_{m,n}=\sum \left(p_{u_{m}}:u_{m}\in U\left(n\right)\right)\\ & 0\leq p_{U(n)}\leq k_{n},k_{n}\in K,\forall n\in N,\\ & 0\leq p_{U(m)}\leq w_{m},w_{m}\in W,\forall m\in M,\\ & {\hat{D}}_{m}\geq q_{m},q_{m}\in Q,\forall m\in M. \end{array}$$

where λ is the coefficient factor ranging between (0,1) and aims at introducing the sub-modular characteristic for sub-channels (resulted from CQI). ${\hat{p}}_{m,n}$ represents the power sum for the *m*th user over the *n*th sub-channel.

The reason of having the piece-wise format is to ensure the allocated user number per sub-channel $r_{n}$ limited to the sub-channel capacity $s_{n}$. Consequently, the GRASP based solver for DPCA is presented in Algorithm 3.

As depicted in Algorithm 3, the first stage (Steps (2)–(6)) of allocation is completed by using a typical greedy strategy (power resources are not segmented into pieces) until the minimum data rate is satisfied for each user. Before the further allocation of the remaining power resources, the power K over sub-channels is divided into segments $\hat{K}$ with a fixed length of $\Delta k_{n}$.
**Algorithm 3** Pseudo-code of GRASP for DPCA  1:Initialization of M, N, K, W, Φ, S, Q, β and ψ. $Dr_{M}=Q$. $Kr_{N}=K$. $Wr_{M}=W$.  2:**while**$\exists Dr_{m}\neq ,\forall m\in M$**do**  3:    $\left[m,n,p\right]=\max(\Delta F_{m,n}\left(\min\left(f^{-1}\left(Dr_{m}\right),k_{n}\right),s_{n}\right))$, ∀n∈N,∀m∈M.  4:    Save and combine *m* to U, *n* to L, and *p* to P. Update $s_{n}$.  5:    $Dr_{m}=Dr_{m}-f\left(p\right)$. $Kr_{n}=Kr_{n}-p$. $Wr_{m}=Wr_{m}-p$.  6:**end while**  7:Discretization of K with fixed interval Δk to form a discrete power set $\Delta k_{n}\in {\hat{K}}_{n}$ among *N* sub-channels.  8:**while** 1 **do**  9:    $RCL\leftarrow R_{m,n}=\left\{\Delta k_{n}\in {\hat{K}}_{n},\forall n\in N,\forall m\in M|\Delta F_{m,n}\left(\min\left(Wr_{m},\Delta k_{n}\right),s_{n}\right)\right\}$.10:    **if** $\sum R_{m,n}\neq 0,\forall n\in N,\forall m\in M$ **then**11:        $c_{max}=\max\left(R_{m,n}\right),R_{m,n}\in RCL$.12:        $c_{min}=\min\left(R_{m,n}\right),R_{m,n}\in RCL$. // the minimum value exclude the value of 0.13:        $\hat{RCL}\leftarrow \left\{R_{m,n}\geq c_{min}+\alpha \left(c_{max}-c_{min}\right):R_{m,n}\in RCL\right\}$.14:        Select an element $R_{m,n}$ from $\hat{RCL}$ at random.15:        $p=\min(Wr_{m},\Delta k_{n})$.16:        Save and combine *m* to U, *n* to L, and *p* to P. Update $s_{n}$.17:        $Wr_{m}=Wr_{m}-p$. $Kr_{n}=Kr_{n}-p$. ${\hat{K}}_{n}={\hat{K}}_{n}-\Delta k_{n}$.18:    **else**19:        Break.20:    **end if**21:**end while**22:**return**L, U and P

The second stage of allocation (Steps (8)–(20)) follows the concept of the construction phase in GRASP. We first evaluate the objective function (11) by ergodic calculation of power segments for all users and sub-channels and save the objective values with user and sub-channel index $R_{m,n}$ as a restricted candidate list (RCL). The RCL is then filtered to reduce the number following $\{R_{m,n}\leq c_{min}+\alpha \left(c_{max}-c_{min}\right):R_{m,n}\in RCL$, where $c_{min}$ and $c_{max}$ correspond to the minimum and maximum values in the RCL set, respectively. The introduced α parameter is to adjust the randomness in generating RCL (the selection turns to be fully random when α=0 and it turns to be fully greedy when α=1). We randomly select a value from the filtered RCL set $\hat{RCL}$, hence the user, sub-channel, and power are allocated.

The termination of the second stage (Step (10)) is reflected by the following three case: (1) full utilization of power resources $\exists Wr_{m}\neq ,\forall m\in M$; (2) sufficient allocation to users $Kr_{n}\neq ,\forall n\in N$; and (3) the user number constraint $r_{n}=s_{n}$ for the second phase allocation.

It is worth noting that, owing to the user capacity constraint, the 0 values in the objective function may lead to the incorrect minimization value of $R_{m,n}$. Therefore, 0 values should be ignored in the calculation (see Step (12)).

The worst-case computational complexity is:

(12) $$\begin{array}{cc} O( & \frac{\left(f^{-1}\left(Q_{max}\right)\right)M^{2}N}{K_{min}}+\frac{MNK_{max}\left(K_{max}N-f^{-1}\left(Q_{min}\right)M\right)}{\Delta K\cdot \min(W_{min},K_{min}-f^{-1}\left(Q_{max}\right))}) \end{array}$$

**Proof.**  Since the proposed GRASP algorithm has two stages, we prove the computational complexity of the two stages separately.

In the first stage, the algorithm meets the minimum power requirement for each user. In the worst-case, the minimum power requirement for one user needs multiple channels to meet. The number of channels to meet the minimum power requirement for each user is $\frac{f^{-1}\left(Q_{max}\right)}{K_{min}}$, which is equal to the number of greedy iterations. In each iteration, there are *N* number of channels as options for each user to select. Therefore, the computational complexity for each user is $\frac{f^{-1}\left(Q_{max}\right)N}{K_{min}}$ and the total computational complexity for all users is $O(\frac{f^{-1}\left(Q_{max}\right)MN}{K_{min}})$.

In the second stage, the algorithm allocates the rest of the resources to users. The maximum total amount of power left for all channels is $K_{max}N-f^{-1}\left(Q_{min}\right)M$. Then, we discretize the remaining power with an interval of ΔK forming $(K_{max}N-f^{-1}\left(Q_{min}\right)M)/\Delta K$ number of power fractions. The maximum number of iterations for each user in the second stage is $(W_{max}-f^{-1}\left(Q_{min}\right))/\Delta K$. Therefore, the computational complexity for the second stage is $O(\frac{\left(K_{max}N-f^{-1}\left(Q_{min}\right)M\right)\left(W_{max}-f^{-1}\left(Q_{min}\right)\right)M}{\Delta K^{2}})$.

The total computational complexity of the algorithm is the sum of the computational complexity of the two stages.    □

### 4.3. Stochastic Sample Greedy Strategy

Given the high-computational loads introduced from the ergodic processing when building the RCL, another heuristic allocation solution, i.e., SSG, is proposed for addressing the DPCA problem. The main concept is applying the stochastic sampling of sub-channels with a probability of ρ to avoid the ergodic searching of sub-channels. Similar to GRASP, the discretization of the power value and the two-stage mechanism are needed for acceleration and maintaining the minimum data rate constraint. The proposed stochastic greedy based solver is presented in Algorithm 4.
**Algorithm 4** Pseudo-code of Stochastic Sample Greedy Algorithm for DPCA  1:Initialization of ρ, M, N, K, W, Φ, S, Q, and β. $Dr_{M}=Q$. $Wr_{M}=W$. Ns=N.  2:**while**n∈N**do**  3:    **if** rand>ρ **then** Ns=Ns\n  4:    **end if**  5:**end while**  6:**while**$\exists Dr_{m}\neq ,\forall m\in M$**do**  7:    $\left[m,n,p\right]=\max(\Delta F_{m,n}\left(\min\left(f^{-1}\left(Dr_{m}\right),k_{n}\right),s_{n}\right))$, ∀n∈Ns,∀m∈M.  8:    Save *m* to U, *n* to L, and *p* to P.  9:    $Dr_{m}=Dr_{m}-f\left(p\right)$. $Kr_{n}=Kr_{n}-p$. $Wr_{m}=Wr_{m}-p$.10:**end while**11:Discretization of K with fixed interval Δk to form a discrete power set $\Delta k_{n}\in {\hat{K}}_{n}$ among *N* sub-channels.12:**while** 1 **do**13:    $\left[m,n,p\right]=\max(\Delta F_{m,n}\left(\min\left(f^{-1}\left(Wr_{m}\right),k_{n},Kr_{n}\right),s_{n}\right))$, $\forall n\in \mathrm{Ns},\forall m\in M,\Delta k_{n}\in {\hat{K}}_{n}$.14:    **if** $\sum \Delta F_{m,n}\left(Wr_{m},k_{n}\right),s_{n})\neq 0,\forall n\in \mathrm{Ns},\forall m\in M$ **then**15:        Save *m* to U, *n* to L, and *p* to P.16:        $Wr_{m}=Wr_{m}-p$. $Kr_{n}=Kr_{n}-p$. ${\hat{K}}_{n}={\hat{K}}_{n}/\Delta k_{n}$17:    **else**18:        Break.19:    **end if**20:**end while**21:**return**L, U and P

Similar to the GRASP algorithm, the constraint of the sub-channel limits and the priority control function are reflected in the objective function (11); the power budget limit from sub-channels is reflected in Steps (11) and (13); and the power budget limit from users is represented in the Step (13).

Owing to the stochastic probability of the sub-channel sampling, the computational complexity can be derived from (12) by replacing *N* with ρN. The complexity of SSG is:

(13) $$\begin{array}{c} O(\rho \frac{\left(f^{-1}\left(Q_{max}\right)\right)M^{2}N}{K_{min}}+\frac{\rho MNK_{max}\left(K_{max}N-f^{-1}\left(Q_{min}\right)M\right)}{\Delta K\min(W_{min},K_{min}-f^{-1}\left(Q_{max}\right))}) \end{array}$$

By comparing the computational complexity in (12) and (13), the SSG reduces the complexity by down-sampling the sub-channels controlled by the ρ factor.

When the resources are abundant for the users, the theoretical performance of the proposed Greedy-based algorithms gets close to that of the Greedy algorithm [38] subject to matroid constraints in the monotone case. According to Buchbinder et al. [39] and [34], the sampling processes in GRASP and Stochastic Sample Greedy Algorithm do not reduce the theoretical approximation guarantee of the original Greedy algorithm [38] for maximizing monotone submodular functions except that the approximation guarantees become stochastic. The theoretical approximation guarantee of the original Greedy algorithm is analyzed through Algorithm 1.

It is worth noting that the applied greedy strategy by using the discrete power resources follows a similar mechanism by using the water-filling concepts. The computation is largely mitigated by using the greedy policy, and the complex constraints can be introduced conveniently. However, the current theoretical performance model is only applicable for the optimization problem with upper bounds, while we consider the lower bound, i.e., the minimum data rate for each user, and user capacity among sub-channels in DPCA. The best performance, expectation value and variance are difficult to estimate theoretically, hence we simulate the allocations and the results are presented in the following section.

## 5. Simulations and Discussions

In this section, we first present a demonstration to reveal the DPCA addressing with the presented stochastic, GRASP, and SSG solutions, respectively. The performance of the presented solutions is evaluated in terms of the data rate and the actual computation time by differing sub-channel numbers and user numbers. Given the trade-off problem existing in configuring parameters in GRASP and SSG, the trade-off analysis is implemented along with the discussions presented for the guidance purpose.

### 5.1. Use Case

We consider a use case of allocating five sub-channels to three users with complicated constraints. The detailed parameter configurations are presented in Table 1. The sub-channel is configured to have 200 kHz bandwidth [40]. The power budget configurations refer to Fang et al. [9], where the power budget for sub-channel is averaged on 30 dBm, and the power budget for users is averaged on 40 dBm. The user capacity per sub-channel is 3. The trade-off factor α is set to 0.8. The power budget is sampled with a fixed interval of 1 dBm. We assume to have five sub-channels for allocation to meet the requirement in Remark 1. The channel propagation model and other environment interference effects are reflected in CQI [9]. We define the concept of the remaining power budget as $W-P_{U}$. The stochastic algorithm, GRASP, and SSG results for DPCA are presented in Table 2.

As depicted in Table 2, all constraints in (5), i.e., power restrictions, minimum data rate, and priority management, are satisfied with the three solutions. The GRASP result outperforms the other two in terms of the remaining power budget and data rate. Specifically the GRASP has the least power budget of 3.2 dBm followed by the SSG of 35.5 dBm and the stochastic algorithm of 42.25 dBm. By calculating the cumulative data rate $\hat{D}$, the GRASP allocation results present the biggest data rate value ($9.22\times 10^{3}$ kbps), which is nearly 1.20 times the stochastic and SSG solutions. Due to the random selection of sub-channels before allocation, the stochastic algorithm and SSG algorithm may keep some sub-channels vacant, contributing to the performance degradation (SSG’s data rate is smaller than GRASP’s for the first user).

### 5.2. Performance Analysis

**Figure 2:**
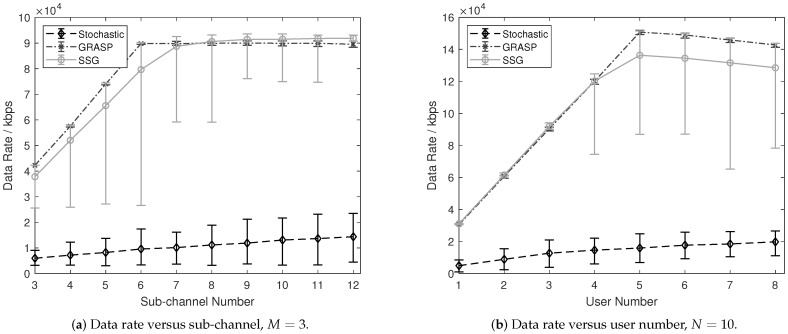
Performance comparison with error bars where α=0.8 and ρ=0.9.

For the further analysis of the proposed solutions, we assume to have homogeneous configurations among sub-channels, such as K=30 dBm, W=60 dBm, S=3, and Q=1000. The elements in CQI are randomly generated following the uniform distribution ranging from [5,6] dB to avoid the unique allocation outcomes. The users are treated equally, hence the priority management is disabled (${\mathrm{flag}}_{\beta }=1$) for the stochastic algorithm, and β=1 for the other two algorithms. By replicating the simulation 200 times, we obtain the averaged cumulative data rates $\hat{D}$ along with error bars for representing the performance uncertainty resulted from the random selection of sub-channels. The performance results with proposed solutions are presented in Figure 2.

As depicted in Figure 2a, the GRASP result is observed to outperform the stochastic solution in terms of showing a larger data rate value and presenting smaller uncertainties (the steady mean data rate of GRASP is 6.26 times that of the stochastic algorithm, while the steady spread is around 0.1 times that of the stochastic one). Compared with SSG, GRASP displays better average values and spreads, when the sub-channel number N≤6 (the increment rate of GRASP is larger than that of SSG by 17%, while the largest spread proportion of SSG is 48 times that of GRASP when N=6). With the further increment of the sub-channel number, when N>7, GRASP and SSG present similar data rates (the absolute error of steady data rate values is limited to $0.2\times 10^{4}$ kbps).

When analyzing the data rate curves among solutions separately, some other results for the stochastic solution are obtained, as shown in Figure 2a. With the increasing sub-channel number *N*, the data rate of the stochastic solution increases slightly (the climb rate is around 0.03 kbps per sub-channel), representing that the stochastic solution maintains a relatively stable performance on average. The spread length of the stochastic solution increases while the increment rate reduces with the further growth of *N*. The low bound of data rate is close to a fixed value attributed to the configured minimum data rate, i.e., $0.3\times 10^{4}$ kbps in this case.

Moreover, the GRASP performance presents stable results with high data rate values and small uncertainties. There is a slight reduction of the data rate when N>6, and we attribute such phenomenon to the non-optimal outcome in the first allocation step. Specifically, the allocated power resources are not sampled when guaranteeing the minimum requirement of transmission. With the increment of the user number, such degradation may be affected more leading to the communication performance degradation.

The averaged data rate curve for SSG grows with the increasing sub-channel number (nearly reaching the peak when N=7) along with the reduction of the increment rate. We can roughly observe that the spreads of SSG are shortened with the growing sub-channel number (the spread is negligible when N=12, i.e., four times M=3). Therefore, we obtain the results that the uncertainty of the SSG solution narrows down (more reliable) with more frequency resources. The performance degradation effect attributed to the randomization in removing sub-channel candidates could be negligible under the spectrum dense environment, i.e., having sufficient sub-channels in this paper.

To analyze the allocation performance on the sub-channel capacity, we varied the user number *M* from 1 to 8, and the data rate results are presented in Figure 2b. Similar to the results in Figure 2a, the GRASP and SSG algorithms outperform the stochastic algorithm for each *M* value. The peak values for GRASP and SSG are obtained when N=5, which means the optimal capacity with N=10 sub-channels is M=5 users, i.e., M=N/2. The reason for having the increasing data rate curves for GRASP and SSG when M<5 is that the current sub-channels are sufficient for allocating a small number of users. Afterward, the data rate decreases because the resources turn to become insufficient with the growing user number. Nevertheless, such reduction is not reflected in the stochastic solution, which means the resources are still not fully exploited by using the stochastic one. The best data rate of SSG is similar to GRASP, while the worst data rates may vary when M≥4. Both GRASP and SSG present close performance in terms of the data rate value and spreads in error bars, representing a similar performance under the resource dense environment (i.e., small user number and large sub-channel number). The averaged data rate curve for SSG grows with the increasing sub-channel number (nearly reaches the peak when N=7) along with the reduction of the increment rate. We can roughly observe that the spreads of SSG are shortened with the growing sub-channel number (the spread is negligible when N=12, i.e., four times M=3). Therefore, we obtain the results that the uncertainty of the SSG solution narrows down (more reliable) with more frequency resources. The performance degradation effect attributed to the randomization in removing sub-channel candidates could be negligible under the spectrum dense environment, i.e., having sufficient sub-channels in this paper.

**Figure 3:**
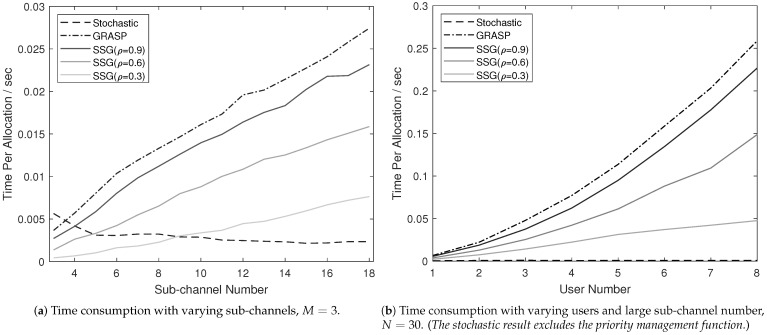
Analysis of actual running time.

We simulated the practical running time for analyzing the computational complexity, where the PC specification was Intel I7-6700 CPU with 16GB of RAM. We set the priority management function into simulation (i.e., β=1,2,3 for GRASP and SSG), and averaged the CPU running time for each allocation iterative in statistics. The time consumption results with diverse channel numbers are presented in Figure 3a. Given the practical running results, we observe that GRASP and SSG consume more time with the increasing sub-channel number *N*, because more sub-channel resources are taken into processing. The time consumption curve for the stochastic algorithm presents a declining tendency. Such decrease results from the fact that there is a higher possibility to meet the constraints with more sub-channels. When comparing the stochastic time consumption curve with the GRASP and SSG ones, it is noticeable that GRASP and SSG have even faster performance than stochastic. Combined with the solution in Figure 2, we conclude that GRASP and SSG are suggested when the allocation is for the extreme spectrum scarce environment (i.e., channel number is very small). By comparing SSG with GRASP, the SSG solution is processed more quickly than that of GRASP for all the *N*. With a smaller ρ value, SSG can be sped up further, although at a cost of losing data rate (the details of losing performance are shown in the following section).

We simulatef the running time consumption with the large sub-channel number (N=30), and the time consumption curves are presented in Figure 3b. It is noticeable that the priority management is disabled in the stochastic algorithm because the stochastic solution is almost impossible to obtain the ordered allocation outcomes with complex constraints when the user number is more than 5. Similar results are shown in Figure 3a, for instance, SSG runs more quickly than GRASP; the small ρ value accelerates the processing in SSG. The time consumption curves for SSG and GRASP in Figure 3a turn to become nearly-linear, while they present an exponential relationship in Figure 3b.

### 5.3. Trade-Off Analysis

**Figure 4:**
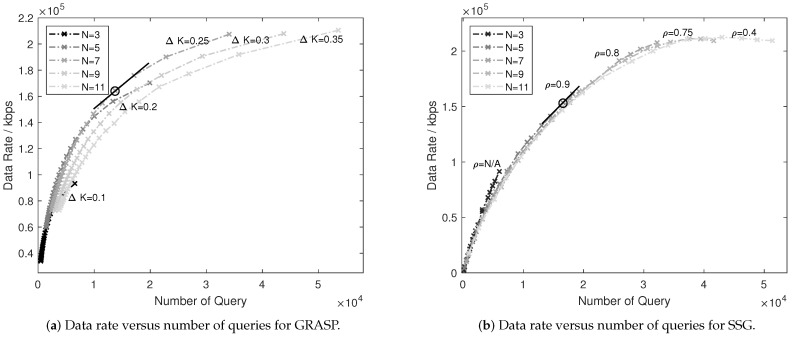
Trade-off balancing for GRASP and SSG, M=3.

Providing that some factors, e.g., ΔK and ρ, are used to balance the trade-off between the performance and the computational complexity in GRASP and SSG solutions, we use the data rate to measure the performance and use the number of query of the objective function (11) to reflect the computational complexity. The reason for using the number of query rather than the actual running time is to improve the accuracy of measurements and reduce the interference effect from other programs. It is worth noting that there is no clear evidence to show that α presents a trade-off effect by simulations, hence the α effect is not discussed in this paper. The ΔK ranges in [0.1,1.5] with the fixed interval of 0.05 and the ρ ranges in [0.1,0.95] with the fixed interval of 0.05. The results of the data rate versus the number of query and sub-channel number *N* are presented in Figure 4.

As depicted in Figure 4a, by increasing ΔK values, data rate and query number rise. We use a fixed straight line (plotted as the solid line) to reflect the consistent selection of the optimal ΔK. Therefore, the optimal ΔK is selected as {0.1,0.2,0.25,0.3,0.35} corresponding to the *N* values in [3,5,7,9,11]. Consequently, we obtain the result that the increasing sub-channel number leads to the relaxation of the sampling step (big ΔK) of power resources and could potentially accelerate the allocation further (the big ΔK corresponds to the smaller power set size). Such acceleration is attributed to the fact that the necessity of dividing power resources into small pieces is released when resources are sufficient. It is also observable that the increment of ΔK tends to be linear along with the growing sub-channel number (ΔK increases by 0.05 when *N* grows by 2). Similar results can be obtained for the SSG algorithm, hence the trade-off curves are not presented.

Moreover, we plot the data rate curve versus the number of query by differing sub-channel numbers and ρ values in Figure 4b. With the same method to select the consistent values in Figure 4a, we observe that, when *N* increases from 5 to 11, the ρ degrades quickly from ρ=0.9 to ρ=0.4. Specifically, only 40% usage of sub-channel resources maintains the ’optimal’ result when N=11, which could significantly improve the allocation efficiency. Combined with the time consumption results presented in Figure 3a, we observe that, when N=11 and ρ=0.3 (approximate to the optimal ρ=0.4), the time consumption is reduced by 66% when comparing the SSG curve and the GRASP curve. In such case, GRASP and SSG present a similar data rate (the optimal points both show the data rate values around $1.5\times 10^{5}$ kbps), which leads to the result that SSG reveals promising potentials under the spectrum dense environment (large sub-channel number). The linear tendency of the curve when N=3 means that it may be difficult to find the optimal ρ under the spectrum scarce environment.

## 6. Conclusions

This paper proposes three heuristic solutions for addressing the DPCA problem in the downlink MC-NOMA system. Multiple complicated constraints are taken into consideration, e.g., the capacity for sub-channels, power budget for sub-channels, power budget for users, minimum data rate, and the priority control during the allocation. All three algorithms are capable of handling complicated constraints. The stochastic algorithm reveals benefits to the large resource allocation scenario without having priority management and the high-performance requirements. GRASP consumes a longer time in processing, while it presents better performance in terms of the data rate and reliability (narrower spread) than the SSG. Considering the large computation scenario, i.e., large user number and sub-channel number, SSG reveals advantages in accelerating the computation at the cost of losing performance. With the sufficient sub-channel number, the computation time is saved by 66% when selecting the optimal configuration of ρ in SSG, with the performance remaining similar. 

## Figures and Tables

**Table 1:** Configuration values.

Para.	Value	Para.	Value
B	200 kHz	K	{28,29,30,31,32} dBm
λ	0.95	β	{1,2,3}
S	{3,3,2,2,2}	W	{38,40,42} dBm
α	0.8	ΔK	1 dBm
CQI	$\left[\begin{array}{ccccc} 4 & 4 & 5 & 6 & 6\\ 6 & 6 & 5 & 4 & 4\\ 6 & 6 & 5 & 5 & 5 \end{array}\right]$	Q	{800,900,1000} kbps

**Table 2:** Demonstration of DPCA allocation for MC-NOMA with heuristic solutions.

Channel		1	2	3	4	5
**Stochastic**	U	{3}	{1,2,3}	N/A	{2,3}	{1,2}
	P dBm	{10.22}	{1.63,23.12,2.74}	N/A	{15.26,10.29}	{11.19,0.31}
**Remaining user budget/dBm**		{25.18,1.32,18.75}				
**Data Rate/kbps**		$\left\{1.80,2.85,3.16\right\}\times 10^{3}$				
**GRASP**	U	{1}	{3,2}	{3}	{2}	{1}
	P dBm	{28}	{12,9}	{30}	{31}	{10}
**Remaining user budget/dBm**		{0,0,0}				
**Data Rate/kbps**		$\left\{17.76,17.91,20.09\right\}\times 10^{3}$				
**SSG**	U	{1}	{3,2,1}	{3}	N/A	{2}
	P dBm	{28}	{12,8,9}	{30}	N/A	{32}
**Remaining user budget/dBm**		{1.0,0,0}				
**Data Rate/kbps**		$\left\{16.33,17.82,20.09\right\}\times 10^{3}$				

## Data Availability

Not applicable.
